# Detail-Preserving Shape Unfolding

**DOI:** 10.3390/s21041187

**Published:** 2021-02-08

**Authors:** Bin Liu, Weiming Wang, Jun Zhou, Bo Li, Xiuping Liu

**Affiliations:** 1 School of Mathematical Science, Dalian University of Technology, Dalian 116024, China; lg_liubin@mail.dlut.edu.cn (B.L.); wwmdlut@dlut.edu.cn (W.W.); 2 College of Information Science and Technology, Dalian Maritime University, Dalian 116024, China; Jun90@dlmu.edu.cn; 3 School of Mathematics and Information Science, Nanchang Hangkong University, Nanchang 330031, China; boli@nchu.edu.cn

**Keywords:** canonical pose, detail preservation, shape deformation

## Abstract

Canonical extrinsic representations for non-rigid shapes with different poses are preferable in many computer graphics applications, such as shape correspondence and retrieval. The main reason for this is that they give a pose invariant signature for those jobs, which significantly decreases the difficulty caused by various poses. Existing methods based on multidimentional scaling (MDS) always result in significant geometric distortions. In this paper, we present a novel shape unfolding algorithm, which deforms any given 3D shape into a canonical pose that is invariant to non-rigid transformations. The proposed method can effectively preserve the local structure of a given 3D model with the regularization of local rigid transform energy based on the shape deformation technique, and largely reduce geometric distortion. Our algorithm is quite simple and only needs to solve two linear systems during alternate iteration processes. The computational efficiency of our method can be improved with parallel computation and the robustness is guaranteed with a cascade strategy. Experimental results demonstrate the enhanced efficacy of our algorithm compared with the state-of-the-art methods on 3D shape unfolding.

## 1. Introduction

The canonical form of 3D shape is very useful for computer graphics applications, such as shape retrieval [1,2,3], shape correspondence [4,5,6] and texture mapping [7,8], as it largely reduces the complexity of 3D shapes caused by various poses. Currently, a popular and commonly used method to obtain canonical forms of 3D shapes is MDS, which comes in classical [9,10], least-squares [11,12,13], and landmark forms [14,15,16,17]. The basic principle of MDS is to maintain the geodesic distances of vertex pairs for the shapes under different poses. To achieve this, it minimizes the sum of squared distance differences between geodesic and Euclidean distances of all vertices pairs on a 3D mesh. However, only satisfying such distance constraints may suffer from serious distortions, leading to a poor accuracy for context-based shape retrieval [3]. A possible way to reduce such distortions is to preserve the local structures of 3D shapes during shape unfolding, that is to make the shape unfolding as rigid as possible [18,19].

In order to preserve as many geometric structures as possible, some researchers tried to obtain feature preserved canonical forms. Most of them first utilized the standard MDS [11] of a shape as guidance, then shape analysis techniques, such as shape deformation based registration [5] or skeleton [20], are applied to prevent distortions with the help of mesh part segmentation [21,22,23]. Recently, Sahillioğlu and Kavan [24] used mass-spring system with non-linear volume constraints to preserve geometric details. Later, Liu et al. [25] introduced an automatic mesh unfolding algorithm by solving a semidefinite programming. The basic idea is to maximize the total variance of the vertex set for a given 3D mesh, while preserving the details by minimizing locally linear reconstruction errors.

However, existing shape unfolding methods have several limitations which hamper their applicability. First, many of them cause significant geometric distortions, as they are the variants of MDS [11], such as the fast MDS [14], non-metric MDS [26] and accelerated MDS [27]. Second, the geodesic distances between all pairs of vertices or landmarks need to be calculated, which is highly time-consuming. Therefore, it is not applicable for high resolution meshes which have hundreds of thousands of vertices. Third, some methods [3,28] have tedious operation processes and their performances highly rely on the accurate execution of each step. Finally, several proposed methods [24,25] need to solve complex non-linear optimization problems which are always time-consuming. In addition, it is difficult to guarantee the robustness of these methods.

Motivated by the above issues, we present a novel mesh unfolding method which has better ability of structure-preserving and ease of use. Our method is based on shape deformation, a technique that can explicitly control the geometric details of meshes. In short, we revisit the mass-spring system model proposed by Sahillioğlu and Kavan [24] and reformulate it in terms of rigid transform energy [19]. Our proposed formulation brings more benefits. Firstly, our model is very simple which only needs to solve linear systems during alternate iteration process. Secondly, it can well preserve the rigidity of the original shape and reduce the geometric distortions as rigid transform energy [19] is considered. Furthermore, our algorithm can be accelerated by using parallel computation and cascade strategy further enhances the robustness of algorithm. In addition, the proposed approach doesn’t need to calculate the pairwise geodesic distances and also has no tedious operation processes, which are timing-consuming and difficult to control.

In summary, the main contributions of our paper are as follows:

- A novel shape unfolding method is proposed for non-rigid 3D mesh based on shape deformation technique. It makes the local deformation be approximately rigid and more details can be preserved.
- The proposed algorithm is easy to implement and parallel computation can be used to improve its computational efficiency. In addition, cascade strategy is used to effectively prevent mesh overstretching.

## 2. Related Work

While canonical form have been improved greatly for the purpose of context-based shape retrieval or other applications, it still has some issues to a certain degree. In this section, we will briefly describe the research progress of canonical form of 3D shape. These works can be classified into two categories, those with details preservation and without. A recent survey of canonical pose can be found in [29]. Afterwards, as the canonical form is one of the special poses, we also investigate some related work about fabrication and beautification.

### 2.1. Shape Unfolding without Detail Preservation

Elad and Kimmel [11] took the pairwise geodesic distance as input to generate the coordinates of all vertices that preserved the specified distances. Two strategies were presented in their paper. One was to calculate the centralized squared geodesic distance matrix and then eigen-decomposition was applied to obtain the resulting shape. The other was to produce the result in a least squares sense by using scaling by majorizing a convex function(SMACOF) to minimise the stress [12]. Unlike the least squares MDS method, which matched the resulting Euclidean distances to the exact geodesic distances, Katz et al. [26] only matched the ordering of distances. However, these algorithms are not applicable in practice for meshes with hundreds of thousands as the computation of geodesic distance is timing-consuming.

The fast MDS method [14] and accelerated MDS method [27] were proposed to improve the calculation efficiency of geodesic distance from different views. The former projected the geodesic distance to Euclidean space one dimension at a time and the latter accurately approximated the pairwise geodesic distance maps through farthest point sampling [30]. However, these methods still need to calculate the pairwise geodesic distance. In addition, the results generated by these methods have significant geometric distortions in Euclidean space, which affect the performances of context-based shape retrieval and shape matching. Rustamov [31] used graph Laplacian matrix, which encoded local geometric and topological properties of a mesh, to generate canonical form of 3D shapes. Dan et al. [32] extend the idea of heat kernel signature to robust isometry-invariant volumetric descriptors for shape retrieval. Similar to the classical MDS method [11], eigenvalues and eigenvectors of the Laplacian matrix in these methods were used to obtain the canonical form, which were called as Global Point Signatures. However, they still suffer from serious geometric distortions. In contrast, our approach applies the local rigid energy which effectively avoids this issue.

### 2.2. Shape Unfolding with Detail Preservation

To preserve more details during shape unfolding, some researchers try to obtain feature-preserving canonical forms. Lian et al. [3] first calculated the standard MDS canonical form for each model. Then the original model was segmented into individual parts by using random walk [21]. Finally, these parts were assembled according to the resulting MDS canonical form. The performance of their method, however, largely depends on the segmentation’s accuracy. Unlike the method proposed by Lian et al. [3], Sahillioğlu [33] utilized volumetric shape deformation technique based on landmarks MDS [15] to preserve initial geometry details. Pickup et al. [28] used the canonical form of mesh skeleton as guidance. They first extracted the curve skeleton [34] from a given model. Then curve skeleton was deformed into a canonical form based on the standard MDS algorithm. Finally, the skeleton driven shape deformation method [20] was used to generate the canonical pose. Nevertheless, the performance of their method depends on the accuracy of skeleton extraction to some extent. In addition, the tedious operation process mentioned above may limit its scope of application in practice.

Some other researchers try to directly obtain the canonical form with details. Pickup et al. [35] calculated the canonical form of a shape by stretching out its limbs. They maximised the Euclidean distances between feature points on the extremities of the mesh while preserving the original edge lengths. Sahillioğlu and Kavan [24] solved this problem by a mass-spring system. This method tried to move each vertex as far away from each other as possible while maintaining the length of finite element. Interior-point method [36] is used to solve their model which is composed of a non-linear objective function and hard volume constrains. In [25], Liu et al. proposed an automatic mesh unfolding method which is solved by semidefinite programming. They first evaluated an approximate pairwise Euclidean distance matrix with unfolding property by maximizing the total variance of the vertex set. Then, the standard MDS or least square MDS [11] was implemented to obtain the final canonical form. However, the above methods only focus on how to preserve edge lengths, but ignore the edge directions of the mesh. In addition, they use sophisticated non-linear optimization algorithm [24,25] which highly affects the applications of their methods. In contrast, our approach imposes constraints on both the edge lengths and directions. Furthermore, the proposed method only needs to solve linear systems and parallel implementation further promotes its practicality.

### 2.3. Other Special Poses

In this section, we discuss the special poses are generated by other objectives, ranging from beatification to fabrication. Symmetrization, for instance, aims to enhance approximate symmetries of an object by computing optimal displacement vectors that pull the shape towards symmetry through a constrained deformation model [37]. In the animation control application, Ref. [38] proposes a fast approach for optimizing parameters such as spring rest lengths so that the artistically modeled shape represents the equilibrium after the mesh has settled under gravity. Refs. [39,40] solve another static equilibrium equation for hair animation based on physically. Ref. [41] optimizes a object into a balanced pose that makes it stand after 3D printing through iterating between carving and deformation. Inverse design methods [42,43] obtain a special resting pose for 3D printing through deforming the mesh into the desired target shape under specified forces when fabricated. Ref. [44] solves sphere spherical surface parameterization for shapes with arbitrary topology based on the concepts of electrostatics.

## 3. Technical Details

In this section we will introduce the details of our shape deformation based unfolding algorithm. The input of our algorithm is a tetrahedral mesh denoted as S, which has *n* vertices and *m* edges. $N_{i}$ is the set of vertices connected to vertex *i*. The embedding of S is defined by the vertex positions $P=\{p_{i}\in R^{3},i=1,2,\ldots ,n$}. Assuming S is deformed into $S^{\prime }$ that has the same connectivity as S and a different geometric embedding $P^{\prime }=\{p_{i}^{\prime }\in R^{3},i=1,2,\ldots ,n$}.

**Figure 1:**
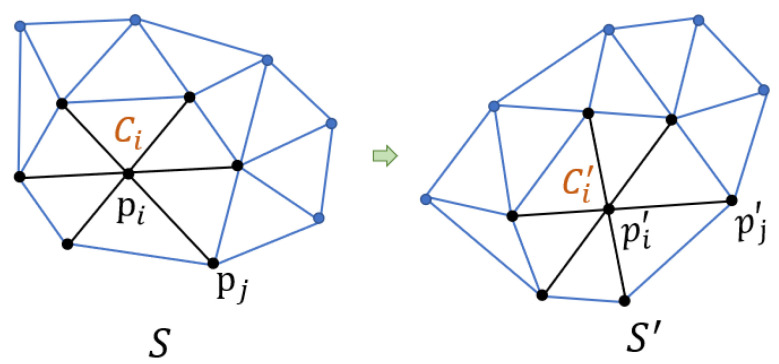
The illustration of local rigid deformation. Cell $C_{i}$ and its deformed version $C_{i}^{{}^{\prime }}$ are composed of these black edges.

It is natural to define the cells among the topological elements of the mesh. In consideration of the required overlap, we choose a vertex-based definition, where each cell is composed of the edges incident upon a vertex, see Figure 1. Afterwards, we could measure the deviation from rigid transformation between two cells in a least squares sense.

### 3.1. Rigid Transformation between Two Cells

Given a cell $C_{i}$ corresponding to vertex *i*, and its deformed version $C_{i}^{\prime }$ (see the black lines in Figure 1), the approximated rigid transformation between them is defined by observing the edges emanating from the vertex *i* in S and $S^{\prime }$. If the deformation is rigid between $C_{i}$ and $C_{i}^{\prime }$, then there exists a rotation matrix $R_{i}$ for all $j\in N_{i}$ such that

(1) $$p_{i}^{\prime }-p_{j}^{\prime }=R_{i}\left(p_{i}-p_{j}\right)\;.$$

If the deformation is not rigid, in the least squares sense, we still could find the best approximating rotation matrix $R_{i}$ by minimizing

(2) $$L\left(C_{i},C_{i}^{\prime }\right)=\sum_{j\in N_{i}}w_{ij}{∥p_{i}^{\prime }-p_{j}^{\prime }-R_{i}\left(p_{i}-p_{j}\right)∥}^{2}\;,$$

where, $w_{ij}$ is the per-edge weight. According to [19], the optimal rotation $R_{i}$ can be easily solved by eigen decomposition. The transformations solved from Equation (2) make sure the local rigidity of S is preserved and mesh details can not be discarded.

### 3.2. Shape Unfolding Model

The proposed shape unfolding model inherits the advantages of mass-spring system. It stretches the distance of non-neighbor vertex pairs on S while preserving the rigidity of each cell. Our model can be written in the following form:

(3) $$\begin{array}{cc} L(P^{\prime }) & =-\sum_{i}\sum_{j\notin N_{i}}k_{ij}{∥p_{i}^{\prime }-p_{j}^{\prime }∥}^{2}\\ \; & +\beta \sum_{i}\sum_{j\in N_{i}}w_{ij}{∥p_{i}^{\prime }-p_{j}^{\prime }-R_{i}\left(p_{i}-p_{j}\right)∥}^{2}\;, \end{array}$$

where, β is a parameter to balance the two items, $w_{ij}$ and $k_{ij}$ are the weights for vertex pairs. Through large experiments, we observe that it obtains satisfactory results when $w_{ij}$ and $k_{ij}$ are set to 1. The influence of β will be discussed in Section 4.

In Equation (3), the first item is applied to stretch the non-neighboring vertex pairs as far from each other as possible. The second item is the summation of Equation (2) for all vertex, which is used to promote a rigid local deformation.

By setting $b_{ij}=R_{i}\left(p_{i}-p_{j}\right)$, the minimization of Equation (3) is equivalent to minimize the following equation

(4) $$\begin{array}{cc} L(P^{\prime }) & =-\sum_{i}\sum_{j\notin N_{i}}∥p_{i}^{\prime }-p_{j}^{\prime }{∥}^{2}+\beta \sum_{i}\sum_{j\in N_{i}}{∥p_{i}^{\prime }-p_{j}^{\prime }∥}^{2}\\ \; & -2\beta \sum_{i}\sum_{j\in N_{i}}{\left(p_{i}^{\prime }-p_{j}^{\prime }\right)}^{T}b_{ij}+\beta \sum_{i}\sum_{j\in N_{i}}{∥b_{ij}∥}^{2}. \end{array}$$

Equation (4) can be further rewritten into the following matrix form

(5) $$\begin{array}{cc} L(P^{\prime }) & =-Tr\left({P^{\prime }}^{T}V_{1}P^{\prime }\right)+\beta Tr\left({P^{\prime }}^{T}V_{2}P^{\prime }\right)\\ \; & -2\beta Tr({P^{\prime }}^{T}H)+F, \end{array}$$

where, Tr(.) represents the trace of a matrix, $V_{1}$, $V_{2}$, H and constant *F* are given by

(6) $$\begin{array}{cc} \; & V_{1}=\sum_{i}\sum_{j\notin N_{i}}{\left(e_{i}-e_{j}\right)}^{T}\left(e_{i}-e_{j}\right),\\ \; & V_{2}=\sum_{i}\sum_{j\in N_{i}}{\left(e_{i}-e_{j}\right)}^{T}\left(e_{i}-e_{j}\right),\\ \; & H=\sum_{i}\sum_{j\in N_{i}}{\left(e_{i}-e_{j}\right)}^{T}b_{ij},\\ \; & F=\beta \sum_{i}\sum_{j\in N_{i}}{∥b_{ij}∥}^{2}, \end{array}$$

where, $e_{i}$ is the ith row of an identity matrix. Taking the derivative of the unknown $P^{\prime }$ and setting the derivative to zero, we can obtain $P^{\prime }$ by solving the following linear system [45]

(7) $$\left(\beta V_{2}-V_{1}\right)P^{\prime }=\beta H\;.$$

## 4. Implementation Details

In general, the proposed algorithm can be solved with the following process: given an initial guess $P_{0}^{\prime }$, the local rotations $\left\{R_{i}\right\}$ are estimated by Equation (2), then $\left\{R_{i}\right\}$ are fixed and $P_{1}^{\prime }$ is obtained by solving Equation (7). The above progress is iteratively performed until an satisfactory solution is generated. This strategy is widely used in computer graphics [19,46]. Our technique can be applied into both triangular mesh and tetrahedral mesh as the data type used in our algorithm is graph structure. However, the representation of tetrahedral mesh obtains the better detail-preserving ability because of the implicit volume constraints. Hence, all experiments are performed on the tetrahedral meshes in our paper. In the following, we will discuss several important parameters used in the optimization.

### 4.1. Initial Exploration

To evaluate the performance of the proposed method for automatic mesh unfolding, we first discuss the effects of parameter β. Intuitively, it affects the local rigidity and the quality of unfolding for a given mesh.

**Figure 2:**
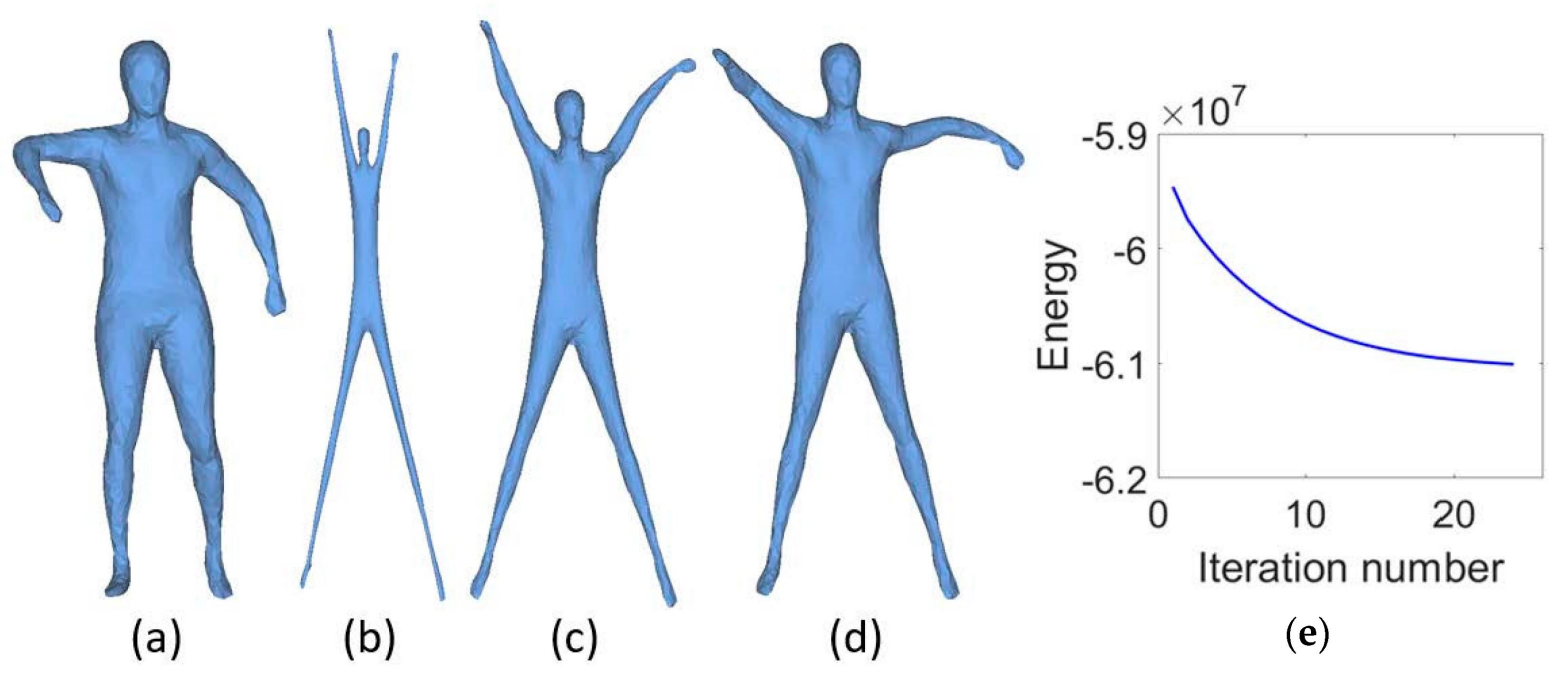
The influences of β. (**a**) is the original mesh. From (**b**–**d**) are the mesh unfolding results with β = 5 × 10${}^{5}$, 1 × 10${}^{6}$, 2 × 10${}^{6}$, respectively. (**e**) shows the convergence curve of the objective function with β = 1 × 10${}^{6}$.

In Figure 2, the Human model (a) is deformed with three different β which are 5 × 10${}^{5}$, 1 × 10${}^{6}$ and 2 × 10${}^{6}$, and the corresponding results are shown in (b), (c) and (d) respectively. Each of them are obtained with 25 iterations. From these results we can observe that small β helps to stretch the mesh, however many details are lost, while large β facilitates the shrinkage of the shape, but it goes against mesh unfolding. The energy curve defined in Equation (3) with β = 1 × 10${}^{6}$ is shown in Figure 2e. We can clearly see that the energy is almost converged after 15 iterations.

**Figure 3:**
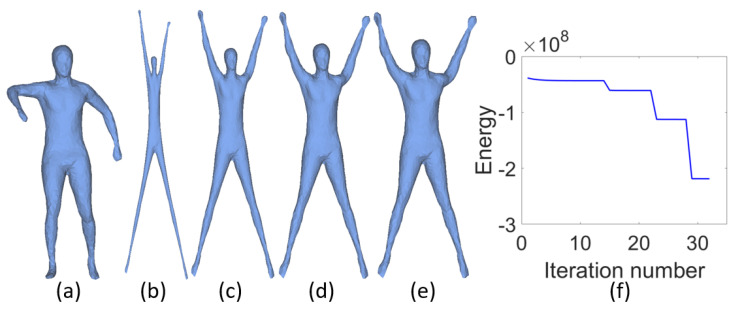
From left to right are the original tetrahedral mesh (**a**), the result of the first cascade (**b**), the result of the second cascade (**c**), the result of the fourth cascade (**d**), the result of the fourth cascade (**e**), and the convergence curve of the objective function (**f**). The jumps in (**f**) are caused by the incremental increasing of β.

**Figure 4:**
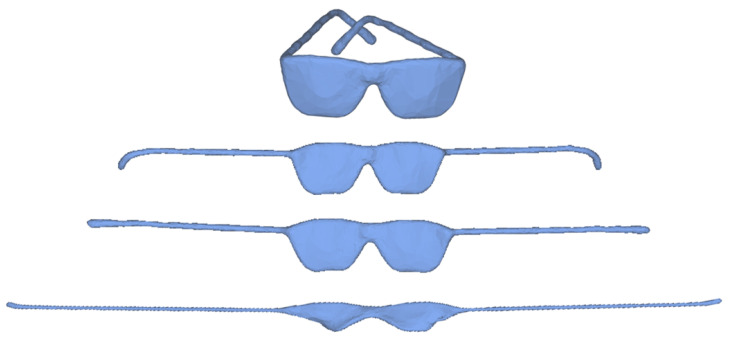
The influences of different initial β. Original glass model (**top**) understretches with β = 10 × 10${}^{6}$ (**second row**), overstretches with β= 1 ×10${}^{6}$ (**fourth row**), and unfolds well with β = 5 × 10${}^{6}$ (**third row**).

From the above discussion we can conclude that choosing a small β results in mesh overstretching in the early iterations and enlarging β is a trade-off strategy during iterations. A straightforward strategy is to increase β per iteration. But it is difficult to design a universal increasing function for β which is suitable for all meshes. Another strategy is to use cascading algorithm with maximum 15 iterations for each cascade. In each cascade, the value of β remains the same and it is increased in the next cascade. Figure 3 illustrates the deformed results with cascading algorithm for the same model shown in Figure 2. The shape in (a) is deformed through four cascades with initial β = 5 × 10${}^{5}$, which is increased two times in each cascade. The corresponding results are shown in (b)–(e) and energy curve is plotted in (f). Experiments demonstrate that the cascading algorithm can obtain better results compared with the fixed value of β. However, cascading algorithm costs more extra running time. Fortunately, the computation efficiency of our model can be improved by parallel computation in each cascade. Figure 4 illustrates the influences of different initial β. We can obtain a series of unfolding results through tuning parameter β, which can meet the different requirements of users.

### 4.2. Parameters

Through extensive theoretical and numerical analysis we finally adopt the following strategies. Each cascade is stopped when it reaches a specified iteration number or the ratio of energy between adjacent iterations below a given precision. In detail, the initial energy $f_{s}$ can be calculated by setting $P^{\prime }=P$, the initial β = 5 × 10${}^{5}$ for most meshes, enlarging the initial β is a good try if the result is not satisfactory, the maximum iterations for each cascade $k_{iter}=15$, the precision $\varepsilon =10^{-4}$ and the maximum number of cascade num=4. In addition, if the ratio $\frac{1}{m}\sum_{i=1}^{m}\frac{l_{i}^{{}^{\prime }}}{l_{i}}\in \left[0.95,1.05\right]$, we also terminate the iteration. Here $l_{i}^{{}^{\prime }}$ and $l_{i}$ are the lengths of the ith edge on the intermediate and original tetrahedral mesh respectively.

Pseudo code of our shape unfolding algorithm is shown in Algorithm 1.

**Algorithm 1** Shape unfolding cascading algorithm
**Input:** Original tetrahedral mesh S, original edge lengths $\left\{l_{i}\right\}$, initial energy $f_{s}$, initial β, the maximum cascading number num, the maximum iterations $k_{iter}$ for each cascade and the precision ε.
**Output:** Deformed tetrahedral mesh $S^{\prime }$.
1:i=1, j=1.2:**while** true **do**3: Get $\left\{R_{i}\right\}$ according to Equation (2).4: Compute intermediate tetrahedral mesh $S^{\prime \prime }$ according to Equation (7) and intermediate edge lengths $\left\{l_{i}^{{}^{\prime }}\right\}$.5: Compute the ratio $\delta =\frac{1}{m}\sum_{i=1}^{m}\frac{l_{i}^{{}^{\prime }}}{l_{i}}$.6: **if** δ∈[0.95,1.05] **then**7:  break8: **end if**9: Compute intermediate energy $f_{e}$ according to Equation (5).10:  **if** $|f_{e}/f_{s}-1|<\varepsilon$ or $i>=k_{iter}$ **then**11:   β=2β, j=j+1, i=0.12:  **end if**13:  **if** j>num **then**14:   break15:  **end if**16:  i=i+1.17:  $f_{s}=f_{e}$18:
**end while**
19: 20:**return**$S^{\prime }=S^{\prime \prime }$.


## 5. Experimental Details

The proposed mesh unfolding method is tested on a non-rigid 3D watertight meshes dataset [47]. This dataset provides a wide range of non-rigid shape classes. Each class contains 20 different poses and each mesh has about 9500 vertices. To improve the computational efficiency, each mesh is first simplified to about 2000 vertices. Then we use tetgen [48] or [49] to generate the corresponding tetrahedral meshes. We obtain about 513 tetrahedral meshes after removing the failed generation. All experiments are performed on a laptop with Intel Core i7−4790K CPU and 32 G RAM.

### 5.1. Quantitative Metrics

To demonstrate the effectiveness of the proposed mesh unfolding method, we quantitatively evaluate the quality of the resulting canonical poses. Specifically, the metrics are defined as

(8) $$E_{rig}=\frac{1}{n}\sum_{i}\sum_{j\in N_{i}}\frac{∥p_{i}^{\prime }-p_{j}^{\prime }-R_{i}\left(p_{i}-p_{j}\right)∥}{∥p_{i}-p_{j}∥},$$

and

(9) $$E_{str}=\frac{2}{n(n-1)}\sum_{i=1}^{n-1}\sum_{j>i}^{n}\frac{d_{ij}^{{}^{\prime }}-d_{ij}}{d_{ij}},$$

where, $E_{rig}$ measures the accuracy of the local rigid approximation, $E_{str}$ measures the mesh stretching between the initial mesh and the canonical pose, $d_{ij}^{{}^{\prime }}$ and $d_{ij}$ are the Euclidean distances between vertex pairs on the deformed and original mesh. From Equations (8) and (9) we can clearly see that an embedding with small $E_{rig}$ and large $E_{str}$ will have good rigidity and strong stretching ability, respectively.

In addition, we also use the following metric to measure the similarity between the pairwise Euclidean distances and the corresponding geodesic distances at the deformed canonical mesh:

(10) $$E_{geo}=∥\frac{D^{{}^{\prime }}}{∥D^{{}^{\prime }}{∥}_{F}}-\frac{G}{{∥G∥}_{F}}{∥}_{F},$$

where, $D_{ij}^{{}^{\prime }}=d_{ij}^{{}^{\prime }}$ is the pairwise Euclidean distance and $G_{ij}^{{}^{\prime }}=g_{ij}^{{}^{\prime }}$ is the corresponding geodesic distance. For each mesh, its value close to 0 imply a good geodesic approximation as the difference is taken between unit matrices. Based on the Equations (8)–(10), we can use the empirical cumulative distribution function (CDF) to evaluate the performance of different algorithms on a whole dataset.

### 5.2. Comparisons

In this section, we compare the results generated by our approach with the state-of-the-art methods: the least squares multidimensional scaling method [11] (LSMDS), skeleton based canonical form [28] (SCF), and detail-preserving mesh unfolding [24] (DPMU). Note that, the method proposed by Liu et al. [25] is not included in our comparisons because of its expensive time consuming, which is 20 times slower than DPMU, and its retrieval accuracy is not the best among the above three methods in the similar dataset [50] from the recent survey paper [29]. To conduct fair comparisons, LSMDS [11] is modified to accept tetrahedral mesh as input and the weights for all vertex pairs are set as 1. For the other methods, all parameters are the same as described in their articles.

**Figure 5:**
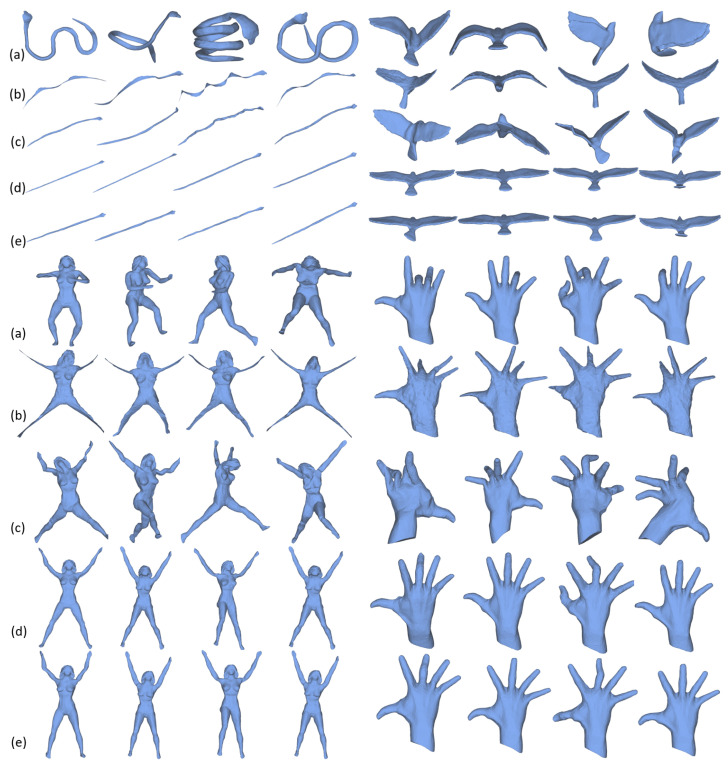
Illustration of mesh unfolding results of different methods. (**a**) original meshes, (**b**) the results of least squares multidimensional scaling method (LSMDS) [11], (**c**) the results of skeleton based canonical form (SCF) [28], (**d**) the results of detail-preserving mesh unfolding (DPMU) [24] and (**e**) our results.

In Figure 5, we illustrate the comparison results among our method, LSMDS, SCF and DPMU. We can observe from this figure that LSMDS always loses some local details of the original meshes, especially for the models with limbs and end-points. While SCF can preserve more details than LSMDS, its efficacy highly depends on the the quality of the skeletal structure which is sensitive to geometry and topology of the mesh. For instance, it is always difficult to extract the accurate skeleton for hand and bird models. As a result, SCF cannot obtain satisfactory results for these meshes. DPMU and our method generate satisfactory results for almost all models. However, our method explicitly preserve the rigidity of the original mesh.

**Figure 6:**
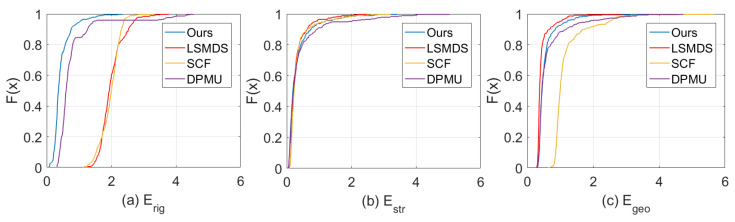
The cumulative probability distributions of $E_{rig}$ (**a**), $E_{str}$ (**b**) and $E_{geo}$ (**c**) for different methods.

Figure 6 shows the CDF curves of $E_{rig}$, $E_{str}$ and $E_{geo}$ for different methods on the dataset [47]. For local rigidity (a), less then 50% samples locate in [0, 2] for LSMDF and SCF methods, and about 95% samples locate in that interval for DPMU, while almost all samples locate in that interval for our method. From (b), we can see that all methods can achieve very similar performances on mesh stretching. In (c), LSMDS can achieve the best performance on geodesic distance preservation, as they consider geodesic distance as hard constraints. However, it is hard to keep the local details. DPMU and our approach have the similar accuracy on this metric and SCF is the worst one. In conclusion, our method is the best for rigid transformation while keeping good performance on $E_{str}$ and $E_{geo}$. Similar results also can be found in Table 1. The difference is that they are calculated on the same category.

**Figure 7:**
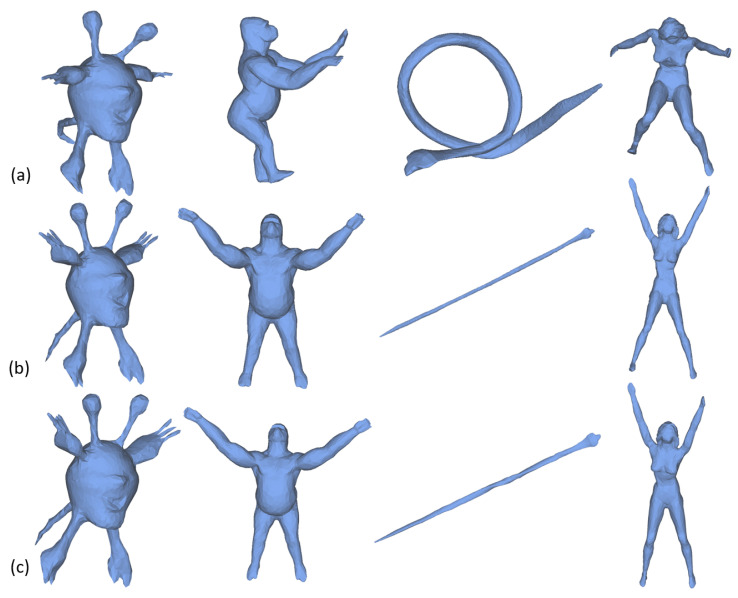
Comparison with state-of-the-art method [24]. (**a**) Original meshes. (**b**) Results of DPMU. (**c**) Our results. The corresponding time consumption is shown in Table 2.

DPMU uses local volume of each vertex as constraint which is nonlinear and the resulting optimization is difficult to solve while the proposed algorithm only needs to solve two simple linear optimization sub-problems. Therefore, our algorithm is faster than DPMU. To prove this, four different shapes are selected and the corresponding unfolding results are shown in Figure 7. In Table 2, we list the running time of our method and DPMU for these shapes. It is clear see from the third and fourth rows that our method consumes less running time for all shapes. In addition, the computational efficiency of our method can be further improved with parallel computation. The last row of Table 2 lists the running time of our method with parallel computation. Note that, the time consumed by the pre-processing process, such as the computation of simplification and tetrahedral mesh which takes about 1.32 s for a shape on the dataset [47], is not taken into account.

### 5.3. Application to Shape Retrieval

In this section, our algorithm is evaluated on non-rigid shape retrieval application [51,52] and compared with the state-of-the-art methods. Two 3D shape retrieval algorithms, which are based on the mean squares error of vertex positions after using optimal rigid transformation (ORT) [53] and the Clock Matching Bag-of-Features (CMBOF) [2], on the original shapes and the unfolding shapes obtained by LSMDS, SCF, DPMU, and Ours. For each strategy, we first centre the mesh, normalize its scale and use a combination of principal component analysis (PCA) [54] and rectilinearity [55] to normalize its orientation. Then, a similarity matrix is saved for online searching based on some statistical measures. We use nearest neighbors (NN) [56] for evaluation in this paper, which calculates the percentage of the closest retrieved shapes that belong to the same class as the query.

Table 3 lists the retrieval accuracy of NN for different methods. From this comparison we can see that our algorithm can achieve comparable shape retrieval performance among the state-of-the-art shape unfolding methods. It is worth noting that only using 3D rigid transformations (ORT) could achieves high accuracy, which is more than 89.5% for all methods. This demonstrates the efficacy of canonical form in shape retrieval.

## 6. Integration with User Control

**Figure 8:**
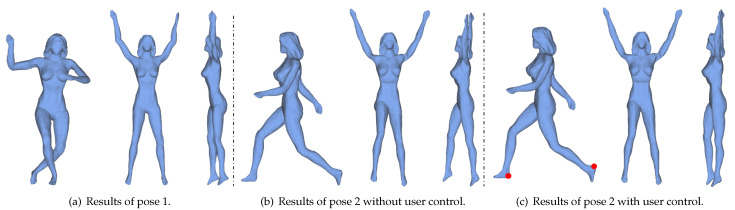
Different poses of the same model ((**a**)-left and (**b**)-left) results in different canonical forms ((**a**)-right and (**b**)-right). With user control (red point pair in (**c**)-left), the quality of the canonical form can be largely improved ((**c**)-right). The second and third columns of each subfigure show the same model under two views.

While our algorithm works well on a large number of models, it is difficult to generate highly uniform canonical forms for models with very different poses. In Figure 8, (a)-left and (b)-left are two poses of the same person. After mesh unfolding, their legs (a)-right and (b)-right have different poses. To solve this problem, an extra item is added in Equation (3) to consider the information provided by users.

(11) $$\begin{array}{cc} L(P^{\prime }) & =-\sum_{i}\sum_{j\notin N_{i}}k_{ij}{∥p_{i}^{\prime }-p_{j}^{\prime }∥}^{2}\\ \; & +\beta \sum_{i}\sum_{j\in N_{i}}w_{ij}{∥p_{i}^{\prime }-p_{j}^{\prime }-R_{i}\left(p_{i}-p_{j}\right)∥}^{2}\\ \; & +\gamma \sum_{(i,j)\in U}u_{ij}{∥p_{i}^{\prime }-p_{j}^{\prime }-t_{ij}∥}^{2} \end{array}\;,$$

where, γ is a parameter to balance the user information, $u_{ij}$ is the weight for vertex pairs, which is also set to 1, U is the control set of vertex pairs and $t_{ij}$ is the control vector specified by user.

Similar to the derivation process of Section 3.2, we can obtain the following linear system

(12) $$\left(\beta V_{2}+\gamma V_{3}-V_{1}\right)P^{\prime }=\beta H+\gamma K\;,$$

where

(13) $$\begin{array}{cc} \; & V_{3}=\sum_{(i,j)\in C}{\left(e_{i}-e_{j}\right)}^{T}\left(e_{i}-e_{j}\right)\\ \; & K=\sum_{(i,j)\in C}{\left(e_{i}-e_{j}\right)}^{T}t_{ij} \end{array}\;.$$

We find that parameter γ has a similar role to β in experiments, so we set γ=β in our algorithm with user control.

In Figure 8, an example is given to show how to specify vertex pairs and control vectors by users. As shown in Figure 8c-left, we first pick one point pair $p_{i}$ and $p_{j}$ on the feet (red nodes), then specify their direction $t_{ij}$ and the length after unfolding with the following strategy. As the intrinsic symmetry plane of this model is perpendicular to z-axis, that is (0,0,1), we restrict $t_{ij}$ to be parallel to (0,0,1) and the length after unfolding to be $\left|\right|p_{i}-p_{j}\left|\right|/2$. The result after solving Formulation (11) is shown in Figure 8c-right which almost has the same pose as the one in Figure 8a-right.

**Figure 9:**
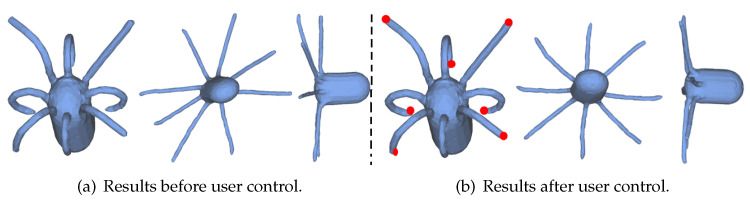
While the canonical form ((**a**)-middle) generated with our method for the Octopus model ((**a**)-left) is satisfactory, its tentacles are not in a plane from the other view((**a**)-right). To improve it, we interactively select points at the end of its tentacles shown as red color in (**b**)-left. The results shown with two views( (**b**)-middle and (**b**)-right), which are obtained by restricting the directions and lengths of the adjacent points.

For the Octopus model shown in Figure 9, our method can give a satisfactory result ((a)-middle). However, its tentacles do not in a plane when viewed from another view ((a)-right). Our algorithm with user control can be used to further improve the quality of the canonical form. We first pick one point at the end of each antenna (red points in (b)-left). Then the directions and lengths between the adjacent antennas are restricted to approximate a regular octagon. The resting results are shown in Figure 9b. We can clearly see that all tentacles are almost in the same plane.

## 7. Conclusions

In this paper, we proposed a novel 3D mesh canonical form generation algorithm based on shape deformation technique. Through extensive experiments, we can see that our method can well preserve the local rigidity of original mesh while unfolding. The non-rigid shape retrieval performance of our method is comparable with the state-of-the-art method. Meanwhile, the proposed algorithm is very simple and easy to implement whose computational performance can be further improved with parallel computation and cascade strategy further enhances the robustness of algorithm. In addition, constraints specified by users can be easily integrated into our approach to further improve the quality of the canonical forms.

**Figure 10:**
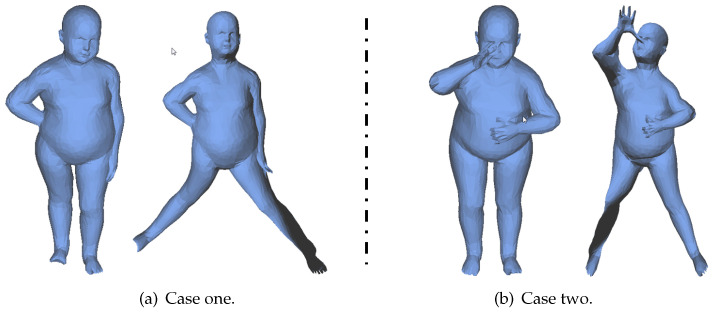
Failure cases. Left: the original mesh. Right: the unfold mesh. Our algorithm unfolds the legs successfully, but the adhesion remains unchanged.

Despite those advantages, the proposed canonical method can not deal with the meshes with topological errors [50,57], where parts of the meshes have been incorrectly fused together or have different genus numbers. As shown in Figure 10, our algorithm unfolds the legs successfully, but the adhesion remains unchanged. In these cases, we may need to manually cut the adhesion parts and then perform our shape unfolding algorithm. In addition, how to choose an adaptive β will be explored in our future work.

## 8. Patents

This section is not mandatory, but may be added if there are patents resulting from the work reported in this manuscript.

## Figures and Tables

**Table 1:** Comparisons on the same category. Numbers in each cell (·,·,·) represent the mean of $E_{rig}$ (left), $E_{str}$ (middle) and $E_{geo}$ (right) for the same category, respectively. For each class, the best is highlighted.

	Category	Ants	Cat	Centaur	Dinosaur	Glasses	Shark
Methods							
LSMDS [11]		(2.25, **0.20**, **0.38**)	(1.53, 0.14, **0.37**)	(1.79, 0.19, **0.33**)	(1.81, 0.22, **0.45**)	(2.04, 0.80, **0.77**)	(1.65, 0.15, **0.38**)
SCF [28]		(2.05, 0.14, 0.89)	(1.80, **0.17**, 0.89)	(2.14, **0.23**, 0.96)	(1.93, 0.22, 1.09)	(2.11, 1.06, 2.05)	(1.86, 0.24, 1.10)
DPMU [24]		(0.54, 0.16, 0.44)	(0.43, 0.11, 0.43)	(0.57, 0.18, 0.38)	(0.61, **0.24**, 0.61)	(2.95, **1.55**, 1.34)	(**0.70**, 0.22, 0.50)
Ours		(**0.38**, 0.16, 0.44)	(**0.32**, 0.11, 0.43)	(**0.32**, 0.15, 0.37)	(**0.33**, 0.19, 0.55)	(**0.51**, 1.01, 1.05)	(0.80, **0.32**, 0.58)

**Table 2:** Runing time of examples in Figure 7 (Unit: second).

Models	Alien	Garilla	Snake	Woman
Num. of Vertices	4062	5091	4229	4823
Time of DPMU	687.05	190.12	1137.23	147.39
Time of ours	84.12	157.01	83.25	115.57
Time of ours (parallel)	30.87	54.37	31.02	46.95

**Table 3:** Retrieval accuracy for different methods on the dataset [47].

	Ori	LSMDS [11]	SCF [28]	DPMU [24]	Ours
ORT	48.2	91.3	89.5	95.1	94.6
CMBOF	-	99.2	99.2	99.5	99.5

## Data Availability

The data presented in this study are available on request from the corresponding author.
